# Application of Electron Paramagnetic Resonance (EPR) Oximetry to Monitor Oxygen in Wounds in Diabetic Models

**DOI:** 10.1371/journal.pone.0144914

**Published:** 2015-12-14

**Authors:** Céline M. Desmet, Aurore Lafosse, Sophie Vériter, Paolo E. Porporato, Pierre Sonveaux, Denis Dufrane, Philippe Levêque, Bernard Gallez

**Affiliations:** 1 Biomedical Magnetic Resonance Research Group, Louvain Drug Research Institute, Université catholique de Louvain, Brussels, Belgium; 2 Endocrine Cell Therapy Unit, Center of Tissue/Cell Therapy, Institut de Recherche Expérimentale et Clinique, Cliniques Universitaires Saint-Luc, Université catholique de Louvain, Brussels, Belgium; 3 Plastic and Reconstructive Surgery Unit, Cliniques Universitaires Saint-Luc, Université catholique de Louvain, Brussels, Belgium; 4 Pole of Pharmacology, Institut de Recherche Expérimentale et Clinique, Université catholique de Louvain, Brussels, Belgium; Martin-Luther-Universität Halle-Wittenberg, GERMANY

## Abstract

A lack of oxygen is classically described as a major cause of impaired wound healing in diabetic patients. Even if the role of oxygen in the wound healing process is well recognized, measurement of oxygen levels in a wound remains challenging. The purpose of the present study was to assess the value of electron paramagnetic resonance (EPR) oximetry to monitor pO_2_ in wounds during the healing process in diabetic mouse models. Kinetics of wound closure were carried out in streptozotocin (STZ)-treated and db/db mice. The pO_2_ was followed repeatedly during the healing process by 1 GHz EPR spectroscopy with lithium phthalocyanine (LiPc) crystals used as oxygen sensor in two different wound models: a full-thickness excisional skin wound and a pedicled skin flap. Wound closure kinetics were dramatically slower in 12-week-old db/db compared to control (db/+) mice, whereas kinetics were not statistically different in STZ-treated compared to control mice. At the center of excisional wounds, measurements were highly influenced by atmospheric oxygen early in the healing process. In pedicled flaps, hypoxia was observed early after wounding. While reoxygenation occurred over time in db/+ mice, hypoxia was prolonged in the diabetic db/db model. This observation was consistent with impaired healing and microangiopathies observed using intravital microscopy. In conclusion, EPR oximetry using LiPc crystals as the oxygen sensor is an appropriate technique to follow wound oxygenation in acute and chronic wounds, in normal and diabetic animals. Nevertheless, the technique is limited for measurements in pedicled skin flaps and cannot be applied to excisional wounds in which diffusion of atmospheric oxygen significantly affects the measurements.

## Introduction

Wound healing is a complex phenomenon that is described schematically by 3 overlapping phases: inflammatory, proliferative and remodeling. During the first phase, inflammatory cells like neutrophils and monocytes are recruited into the wound to prevent infections and to clean the wound from dead tissue and other foreign bodies. The next step is characterized by the proliferation of cells to form new tissue: fibroblasts and endothelial cells (neovascularization) that form granulation tissue; and keratinocytes that proliferate and migrate to restore the epithelium (reepithelialization). The last step is the remodeling of the extracellular matrix that increases the wound tensile strength [1, 2]. In an acute wound, the sequence of these events is well represented and results in the restoration of anatomic and functional integrity of the tissue [3]. But, on the contrary, chronic wounds like diabetic ulcers, venous ulcers and other pressure sores [4] fail to restore the integrity of the tissue due to a dysregulation of this well-orchestrated process [3]. Etiologies of chronic wounds are multifactorial but one common factor observed in impaired wound healing is tissue hypoxia [4, 5].

Oxygen plays an important role at the several levels of the wound healing process [5–7]. First, during the inflammatory phase, oxygen is used by NADPH oxidases to produce reactive oxygen species (ROS) for oxidative burst [8]. During the proliferative phase, oxygen is used for oxidative phosphorylation in order to produce sufficient energy for proliferating cells with high metabolic need. Finally, oxygen is necessary for mature collagen production and deposition by fibroblasts during proliferative and remodeling phases. Indeed, hydroxylation of proline and lysine residues of procollagen chains is necessary to stabilize the triple helix of collagen. This reaction is catalyzed by hydroxylases and requires oxygen, iron, ascorbic acid and α-ketoglutarate as cofactors [9]. Consequently, prolonged hypoxia can be deleterious at each phase of the wound healing process: wounds suffering from poor oxygenation typically present a sustained inflammatory phase, a prolonged proliferative phase and/or a poor wound tensile strength [6].

While oxygen is known to play a key role in wound healing, there are only few experimental studies aimed at measuring oxygen in acute wounds and none in chronic wounds. The reason is that, to date, no method has been validated to measure pO_2_ non-invasively, reliably and repeatedly in a wounded tissue. The use of polarographic Clark electrodes is the gold standard method to measure tissue oxygenation [6] but has several drawbacks. This method is invasive and the introduction of electrodes by itself modifies tissue environment by inducing injuries. Also, these electrodes consume oxygen during the measurements. Furthermore, reliable longitudinal studies of oxygenation are impossible because electrodes cannot be placed exactly at the same location on consecutive days. Hunt and his group, who were the first to study oxygenation in wound healing, developed subcutaneously implanted wire mesh cylinders and adapted subcutaneous tonometers for oxygen tension measurements in wounds [10]. The first technique is based on measurement of oxygen tension in the wound fluid aspirated from the dead wound space created by implantation of cylinders in the dorsal skin of animals [11, 12]; the second consists in the measurement of oxygen in the effluent of deoxygenated Ringer’s lactate or saline perfused within the subcutaneously implanted tonometer. However, these techniques also suffer from some disadvantages. Implantation of cylinders or tonometers is complex and invasive and measurements in the aspirated wound fluid can easily be influenced by air bubbles contamination. More recently, the luminescence technique was used to image wound pO_2_. Using films containing luminescent optical oxygen sensor, maps of surface wound pO_2_ were obtained by Schreml and his group in human wounds [13, 14] whereas Li *et al*. measured transdermal pO_2_ in burn wounds and skin grafts in pigs with oxygen-sensing paint-on bandages [15].

EPR oximetry could be a promising technique to measure wound pO_2_ as it overcomes principal disadvantages of the methods previously cited. EPR oximetry is a technique based on the paramagnetic properties of oxygen. The interaction between unpaired electrons of oxygen and some paramagnetic probes, such as lithium phthalocyanine (LiPc), shortens the T_2_ relaxation time which is related to a broadening of the EPR spectrum recorded from the probe. Line width broadening is directly proportional to the surrounding oxygen concentration, so that with appropriate calibration tissue pO_2_ can be determined from the line width of the spectrum recorded from the paramagnetic oxygen sensor previously implanted in the tissue [16–19]. This technique presents the advantage to be minimally invasive: once the oxygen sensor is implanted in the tissue, EPR spectra can be recorded non-invasively *in vivo* using a low frequency (L-band) EPR spectrometer [17, 18]. Measurements can be recorded at the same location repeatedly during a long period of time [18] up to at least 5 years [20], allowing longitudinal studies. Moreover, the technique does not consume oxygen contrarily to microelectrodes and, consequently, it does not modify the environment where the pO_2_ is measured. EPR oximetry was previously used to monitor oxygenation in several types of tissues [18] such as brain [21–28], heart [29–34], gastrointestinal tract [35], skeletal muscle [36–38], liver [39–45], kidneys [46] and skin [47–49], to characterize the tumor microenvironment [50, 51], to measure oxygenation in ovarian grafts [52] and bone fractures [53–55]. Although EPR oximetry may be beneficial for studying the implication of oxygen during the healing process [56], to the best of our knowledge only 2 publications in the scientific literature have reported such application [57, 58]. To date, EPR oximetry has never been applied in the context of skin wound healing in diabetic models.

Therefore, the aim of this study was to assess the value of EPR oximetry to follow pO_2_ variations during wound healing in diabetic models. For this purpose, we first validated the use of a murine diabetic model as an impaired wound healing model. Second, EPR oximetry was applied to two different types of wounds, a full-thickness excisional skin wound and a pedicled skin flap, and oxygenation was monitored during the healing process.

## Materials and Methods

Two types of diabetic models were investigated: type 1 diabetes induced by streptozotocin (STZ) in NMRI mice and type 2 diabetes using genetically modified diabetic BKS(D)-Lepr^db^/JOrIRj (db/db) mice. For non-diabetic controls, untreated NMRI mice or control db/+ mice were used. Two types of wound models were investigated: an excisional full-thickness skin wound and a pedicled skin flap. In each model, tissue oxygenation was monitored before and after surgery using lithium phthalocyanine (LiPc) crystals as oxygen sensor.

### Animals

All animals used in this study were obtained from Elevage Janvier (France). Mice were housed individually in a temperature-controlled facility with a 12-hour dark/light cycle and received water and normal food ad libitum during all the experiment. All animal experiments were approved by the local Ethical Committee for animal care of the Université catholique de Louvain (authorization #2010/UCL/MD/01).

### Diabetic models

#### Type 1 diabetes

Male 5 to 6-week-old NMRI mice received an intraperitoneal injection of 30 mg/kg of streptozotocin (STZ) (Sigma-Aldrich, Belgium) dissolved in citrate buffer (0.1 M, pH 4.5) during 5 consecutive days to induce a type 1 diabetes. The diabetic state was confirmed by measuring non-fasting blood glucose (NFBG) once a week until surgery. Blood was sampled from the tail vein and analyzed with a GlucoMen LX Plus glucometer (Menarini Diagnostics, Italy). Animals were considered diabetic when NFBG was >250 mg/dL. Three groups of diabetic mice were included in the study: mice presenting NFBG >250 mg/dL for 4 weeks (STZ-4w), 5 weeks (STZ-5w) or 7 weeks (STZ-7w). For non-diabetic controls, untreated 9-week-old male NMRI mice were used.

#### Type 2 diabetes

Male BKS(D)-Lepr^db^/JOrIRj (db/db) mice were used as a model of type 2 diabetes. These mice develop hyperphagia, obesity, hyperglycemia and hyperinsulinemia [59] due to an autosomal recessive mutation in the gene encoding the leptin receptor [60]. NFBG was controlled weekly (first measurement in 5-week-old mice) until surgery. Male age-matched control heterozygous littermates db/+ mice, that do not express the diabetic phenotype, were used as control non-diabetic mice.

### Wound models

#### Full-thickness excisional skin wound model

The hair of the back of mice was shaved with an electric shaver before surgery. A full-thickness excisional skin wound was created on the dorsal midline with a 6 mm round skin biopsy punch (Kai Europe GmbH, Germany) during ketamine (80 mg/kg)/xylazine (8 mg/kg) anesthesia. A dressing was fixed on the wound with 6–0 sutures. Wound surgery was performed on STZ-treated diabetic mice, untreated non-diabetic NMRI mice, db/db mice and control db/+ mice.

#### Pedicled skin flap model

The hair of the back of mice was shaved prior to surgery. A U-shaped 30 x 8 mm pedicled skin flap was raised on the back of db/db mice and control db/+ mice during ketamine (80 mg/kg)/xylazine (8 mg/kg) anesthesia and stitched with 6–0 sutures. Pedicled flaps were made with a narrow pedicle (low ratio pedicle/length) to induce a hypoxic challenge at the distal part of the raised flap.

### Kinetics of wound closure

Kinetics of closure of excisional full-thickness skin wounds were measured in healthy NMRI, STZ-4w, STZ-5w, STZ-7w, 12-week-old db/db and 12-week-old db/+ mice. Kinetics of closure were determined by measuring the wound diameter using a caliper during the healing process.

### EPR oximetry

#### Implantation of LiPc crystals

Lithium phthalocyanine (LiPc) crystals, kindly provided by H.M. Swartz (Dartmouth Medical School, Hanover, NH, USA), were used as the oxygen-sensitive sensor [61]. They were placed in the tissue as follows:

Model 1—oxygen sensor in the center of excisional full-thickness wounds: LiPc crystals were placed with a 26 G needle in the center of the wounds, under the fascia, in untreated NMRI mice and 12-week-old db/db mice during wound surgery.

Model 2—oxygen sensor in the edges of excisional full-thickness wounds: After shaving of the back of 11-week-old db/db mice, LiPc crystals were placed in the healthy subcutaneous tissue using a 26 G needle under ketamine (80 mg/kg)/xylazine (8 mg/kg) anesthesia, 7 days before wound surgery.

Model 3—oxygen sensor in pedicled flaps: 7 to 10 days before wound surgery, the hair of the back of 11-week-old db/db and control db/+ mice was shaved and LiPc crystals were implanted subcutaneously using a 26 G needle under ketamine (80 mg/kg)/xylazine (8 mg/kg) anesthesia. LiPc crystals were placed on the dorsal midline at 2 sites separated by 10 mm in order to be at the proximal (15 mm from the pedicle) and distal (25 mm from the pedicle) parts of the raised flap.

#### EPR oximetry measurements

An EPR spectrometer (Magnettech, Berlin, Germany) equipped with a low-frequency microwave bridge operating at 1.2 GHz and an extended loop resonator (with 1-cm depth sensitivity) was used for pO_2_ measurements. Animals under gaseous anesthesia (isoflurane, 2%), to preserve tissue oxygenation [62], were positioned in the EPR spectrometer, the region containing LiPc crystals placed under the surface coil. The temperature of mice was maintained to 37°C during measurements. Spectra were recorded with a modulation amplitude less than one third of the peak-to-peak line width. Parameters of acquisitions were the following: sweep field: 0.1 mT; sweep time: 20 to 30 s; number of points: 512–1024; number of scans: 5 to 20; modulation frequency: 10 kHz; modulation amplitude: 0.001875 to 0.00375 mT; power: 7.70–10.40 mW; gain: 200. The pO_2_ of the tissue was determined by converting the peak-to-peak line width of the recorded EPR signal with a calibration determined previously for the same batch of LiPc crystals used in the present study (S1 Fig). Calibration was determined by flushing LiPc crystals at pO_2_ ranged from 0 to 159.6 mmHg (0 to 21% O_2_). Nitrogen and oxygen were mixed using an Aalborg gas mixer (Orangenburg, NY, USA) and the oxygen content was determined using a Servomex oxygen analyzer OA540 (Analytic Systems, Brussels, Belgium). EPR spectra were recorded using a Bruker EMX EPR spectrometer (Rheinstetten, Germany) operating at 9.5 GHz (X-band) and equipped with a temperature controller BVT-3000. Measurements were realized at 37°C. Parameters were the following: center field = 337.63 mT; sweep width = 0.3 mT; power = 0.1 mW; gain = 632; modulation frequency = 10 kHz; modulation amplitude = 0.001 mT; time constant = 5.12 ms; conversion time = 40.96 ms; sweep time = 20.97 s; 512 points; 3 scans.

The pO_2_ was monitored repeatedly during the healing process. Control pO_2_ values of healthy subcutaneous tissue were obtained by measuring repeatedly the pO_2_ in unwounded non-diabetic and diabetic mice.

Response to pO_2_ variations was verified one month after implantation of the oxygen sensor by a carbogen (95% O_2_ and % CO_2_) breathing challenge (hyperoxia) and after the animal sacrifice (acute hypoxia).

### Intravital microscopy

Intravital microscopy was performed on db/+ and db/db mice as previously described [63]. Mice were shaved on the day before surgery. Animals were anesthetized with ketamine (80 mg/kg)/xylazine (8 mg/kg) and a two-sided titanium frame possessing a circular 12-mm opening was implanted on the dorsal skin flap of the mice. The opening was centered on a size-matched area where superficial skin and both the upper and lower fascia had been surgically removed. The titanium frame was sutured and the opening was closed with a circular glass coverslip secured with a snap ring. Fluorescein 5’-isothiocyanate (FITC) dextran (Sigma Aldrich, Belgium) was injected in the tail vein 10 minutes before imaging the skin microcirculation. Images were then captured using a CCD camera connected to an inverted Axiovert microscope (Zeiss, Germany).

### Statistical analysis

Results were expressed as mean ± standard error of the mean (SEM).

One-way ANOVA followed by Dunnett’s multiple comparison post hoc test or unpaired t-test was used to compare wound diameter between groups where appropriate.

For the comparison of pO_2_ values at day 2 in excisional wounds *vs* basal pO_2_ in unwounded tissue, unpaired t-test was used both for db/db and NMRI groups.

One-way ANOVA with Dunnett’s multiple comparison post hoc test was used to compare wound pO_2_ between groups in the flap model. P values <0.05 (*), <0.01 (**) and <0.001 (***) were considered to be statistically significant. Statistical analyses were performed using GraphPad Prism 5 (GraphPad Software, La Jolla, CA, USA).

## Results

### Selection of diabetic animal model

Two types of diabetes were investigated to evaluate if diabetes induces impaired healing in mouse models. Non-fasting blood glucose (NFBG) was monitored to verify the diabetic state. When diabetes was verified, kinetics of wound closure were monitored to evaluate if diabetic models presented impaired wound healing.

#### Non-fasting blood glucose

**Fig 1:**
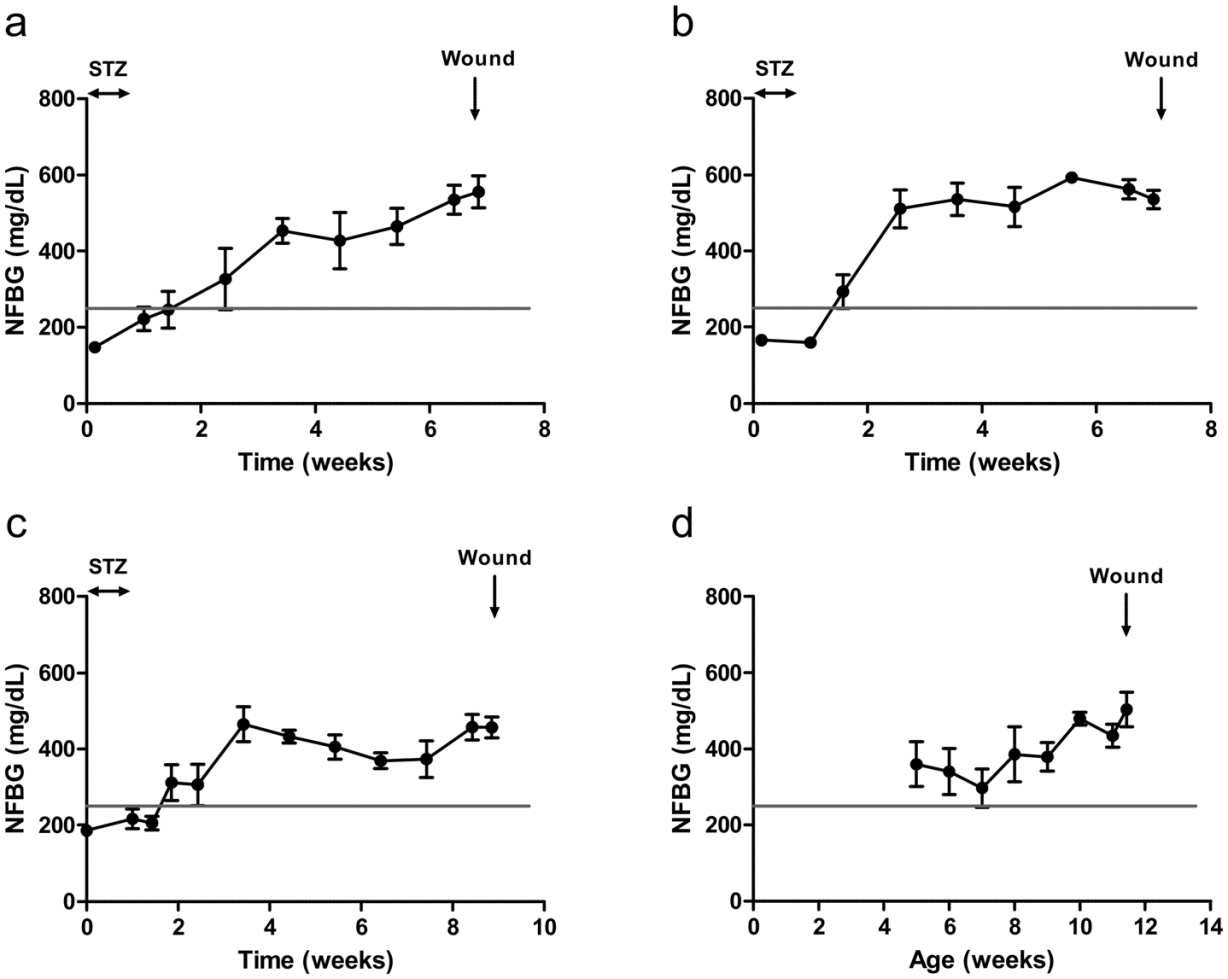
Confirmation of diabetic state before wounding. NFBG was monitored weekly in STZ-induced diabetes after 4 weeks (a), 5 weeks (b) and 7 weeks (c) and in genetically modified db/db mice (d). Mice were considered to be diabetic when NFBG >250 mg/dL. Results are expressed as mean ± SEM (n = 4).

Non-fasting blood glucose (NFBG) was monitored weekly in STZ-induced and db/db mice to confirm the diabetic state before wounding. In the STZ-induced diabetic group, NFBG was 168 ± 8 mg/dL prior to STZ treatment. Mice injected with STZ became hyperglycemic (NFBG > 250 mg/dL) 5 to 12 days after the last STZ injection. Then, NFBG remained above 250 mg/dL until excisional full-thickness skin wounding was performed 4 (STZ-4w), 5 (STZ-5w) or 7 weeks (STZ-7w) later (Fig 1). At the time of wounding, NFBG was 556 ± 42 mg/dL, 536 ± 24 mg/dL and 457 ± 27 mg/dL, respectively, for STZ-4w, STZ-5w and STZ-7w mice. Db/db mice had a NFBG above 250 mg/dL during the 7-week follow-up before wounding. At the time of surgery, 12-week-old db/db mice had a NFBG of 504 ± 45 mg/dL. Non-diabetic control db/+ mice had a NFBG of 120 ± 16 mg/dL at wounding time.

Diabetes was thus confirmed in all diabetic mouse models at the time of surgery.

#### Wound closure kinetics in the different diabetic models

**Fig 2:**
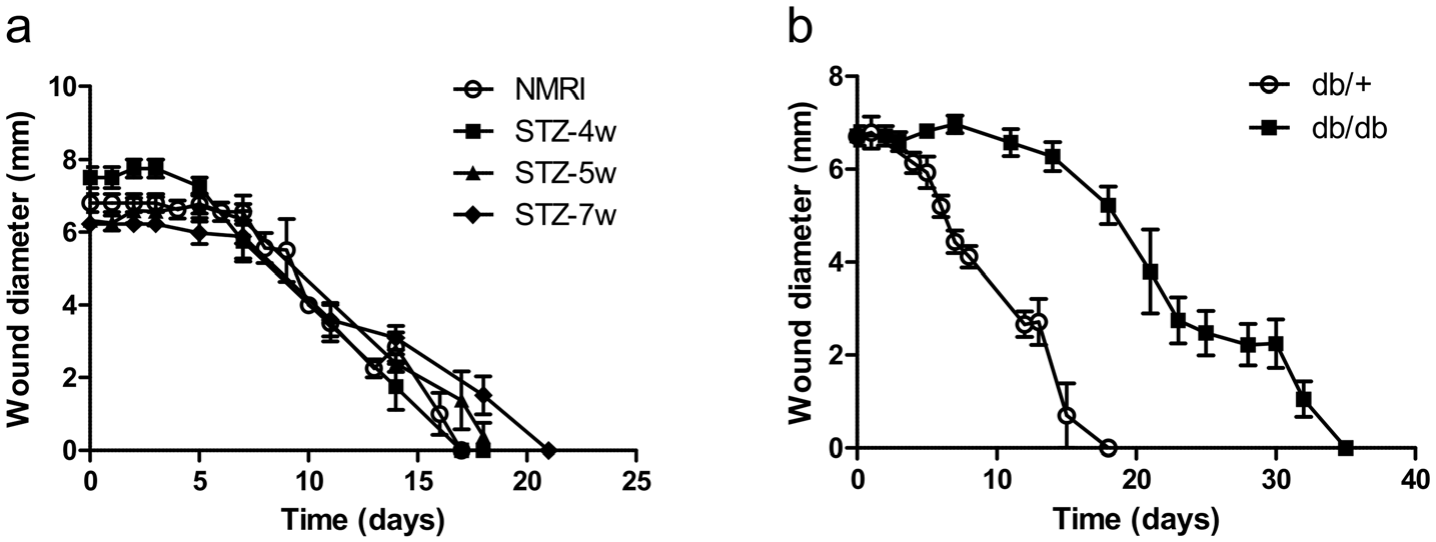
Db/db mice exhibit delayed excisional wound closure. Kinetics of wound closure were monitored in STZ-induced diabetic mice *vs* control NMRI mice (a) and db/db mice *vs* control db/+ mice (b). Results are expressed as mean ± SEM (n = 4–6).

In order to determine which type of diabetic murine model was the most relevant for this study, the kinetics of closure of excisional full-thickness skin wounds were quantified in the different models by measuring the wound diameter during the healing process. No difference was observed in the kinetics of closure of excisional full-thickness skin wounds in the STZ-4w, STZ-5w and STZ-7w groups compared to the control non-diabetic group (Fig 2a). In contrast, complete wound closure was highly significantly delayed in db/db mice (full closure at 35 days) compared to db/+ mice (full closure at 18 days) (Fig 2b).

**Fig 3:**
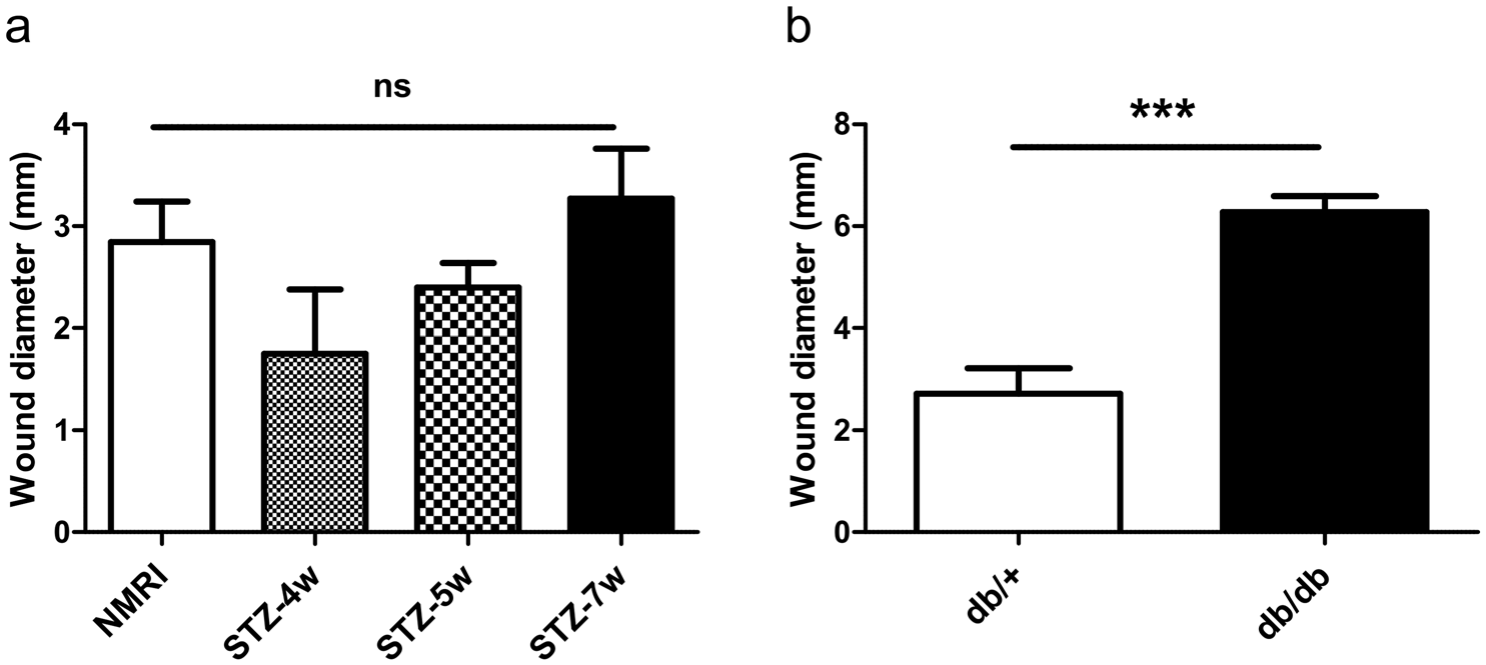
Db/db mice exhibit impaired wound healing. Wound diameter at day 14 in STZ-induced diabetic mice *vs* control NMRI mice (a) and db/db mice *vs* control db/+ mice (b). Results are expressed as mean ± SEM (n = 4–6). ns: non significant (One-way ANOVA and Dunnett’s multiple comparison post hoc test, comparison with control NMRI mice); ***: p<0.001 (unpaired t-test).

At day 14, no significant difference was observed in the wound size between STZ-4w, STZ-5w, STZ-7w and control non-diabetic mice (Fig 3a), whereas the wound size was significantly larger in db/db compared to db/+ mice (6.28 ± 0.31 mm *vs* 2.72 ± 0.50 mm; p < 0.001) (Fig 3b). Consequently, db/db mice were selected as the model of impaired wound healing for the oxygenation study.

### EPR oximetry

#### Basal pO_2_ values in the unwounded subcutaneous tissue

In the unwounded subcutaneous tissue, a mean pO_2_ value of 18.8 ± 1.2 mmHg was measured in non-diabetic NMRI mice. In diabetic mice, the mean pO_2_ value was 20.7 ± 1.2 mmHg. No significant difference was observed between the basal pO_2_ in non-diabetic and diabetic mice (unpaired t test, p = 0.31). The pO_2_ was stable during a 1-month monitoring period.

**Fig 4:**
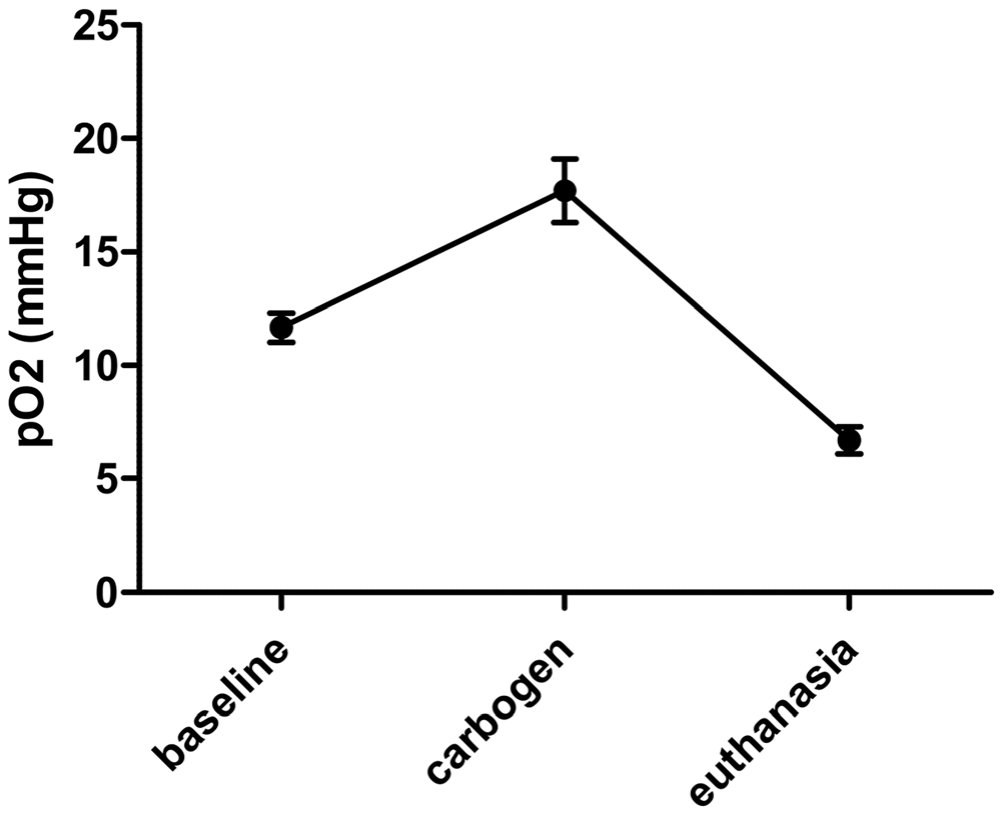
LiPc crystals responded to pO_2_ variations one month after the implantation in the skin tissue. Typical experiment realized on a mouse 1 month after implantation of the LiPc crystals in the tissue. Results are expressed as mean ± SEM (n = 3–4).

Also, we observed that the response to pO_2_ variations was preserved one month after LiPc implantation (Fig 4).

#### Model 1: oxygen sensor in the center of excisional full-thickness wounds

**Fig 5:**
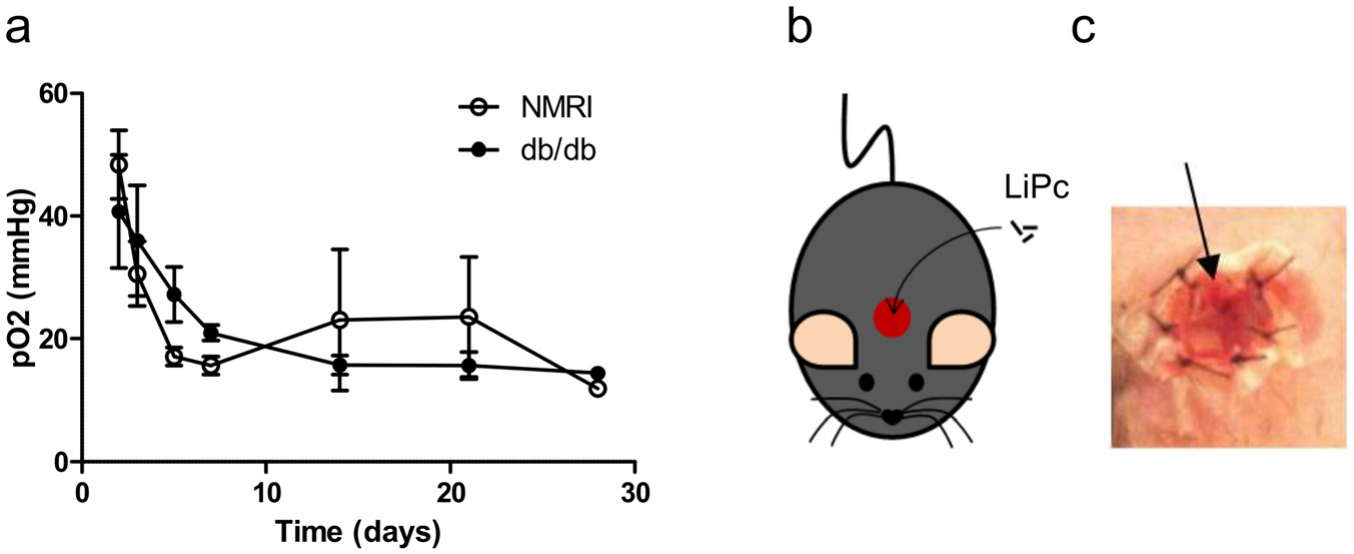
High pO_2_ is observed at the center of full-thickness excisional skin wounds early after wounding. LiPc crystals were implanted at the center of excisional wounds in control NMRI and diabetic db/db mice during the surgery (b, c) and pO_2_ was monitored repeatedly by EPR oximetry during wound healing. Results are expressed as mean ± SEM (n = 4) (a).

In the center of excisional full-thickness skin wounds (Fig 5b and 5c), high pO_2_ values were observed at day 2 similarly in diabetic (40.8 ± 9.2 mmHg) and control non-diabetic (48.4 ± 5.6 mmHg) mice. These values were significantly higher than those measured in the unwounded subcutaneous tissue in both diabetic (20.7 ± 1.2 mmHg, p = 0.012, unpaired t-test) and non-diabetic (18.8 ± 1.2 mmHg, p = 0.009, unpaired t-test) mice. In this configuration, the oxygen sensor located at the center of wounds was in direct contact with surrounding air. After 7 days, the pO_2_ decreased regularly to reach values normally observed in the healthy subcutaneous tissue (Fig 5a). At this time, the LiPc sensor was trapped in the neo-formed granulation tissue of wounds.

#### Model 2: oxygen sensor in the edges of excisional full-thickness wounds

**Fig 6:**
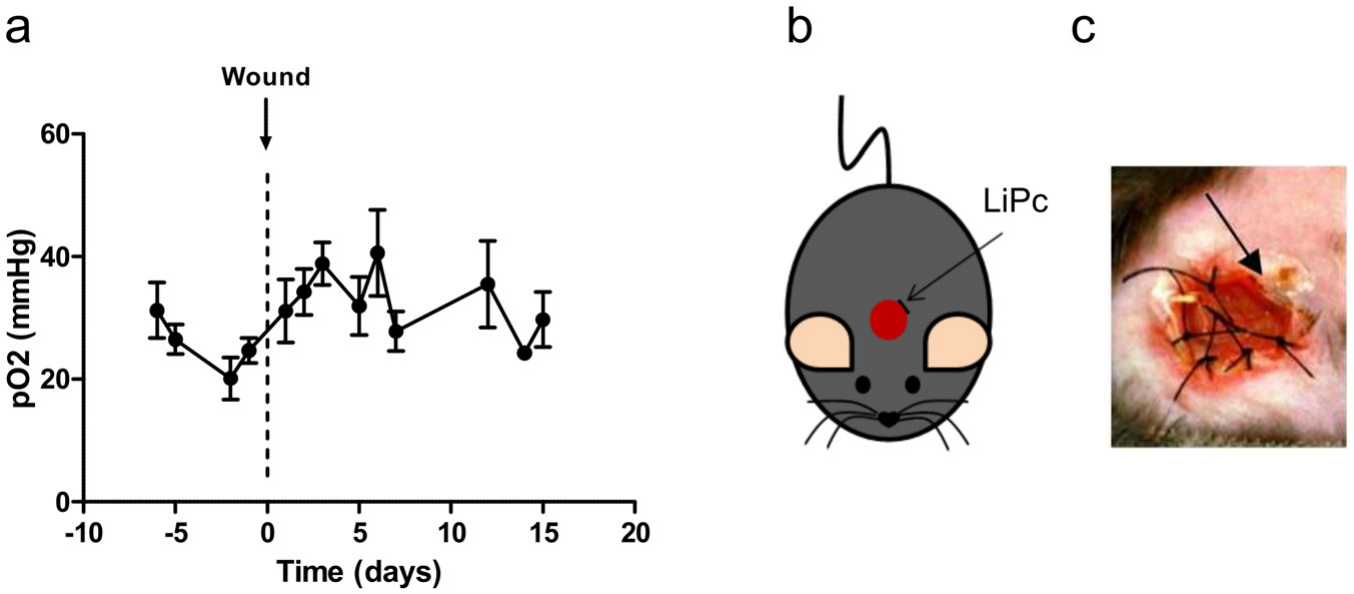
The periphery of full-thickness excisional skin wounds does not exhibit hypoxia. LiPc crystals were implanted 7 days prior to surgery at the periphery of diabetic excisional wounds (db/db mice) (b, c) and pO_2_ was monitored repeatedly by EPR oximetry during wound healing. Results are expressed as mean ± SEM (n = 4) (a).

With sensors at the edges of excisional wounds (Fig 6b and 6c), basal pO_2_ value 1 day prior to surgery was 24.7 ± 2.1 mmHg consistent with values obtained in the subcutaneous healthy tissue. A pO_2_ increase was observed from day 1 to day 3. At day 15, pO_2_ was decreased to recover normal basal values (Fig 6a).

#### Model 3: oxygen sensor in pedicled skin flaps

**Fig 7:**
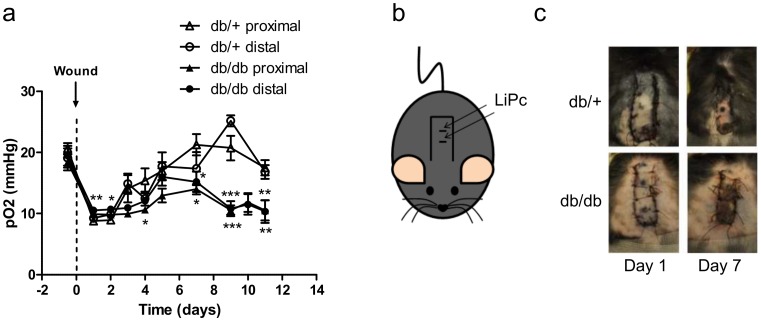
Pedicled skin flaps exhibit prolonged hypoxia in diabetic db/db mice contrarily to control db/+ mice. LiPc was implanted in the back of diabetic db/db (n = 14) and non-diabetic db/+ mice (n = 12) (b, c) 7 to 10 days prior to surgery. The pO_2_ measurements by EPR oximetry were realized at distal and proximal regions of the flaps. Results are expressed as mean ± SEM (a). *: p <0.05, **: p<0.01 and ***: p<0.001 (One-way ANOVA and Dunnett’s Multiple Comparison post hoc test, comparison with db/+ proximal).

In the flap model, LiPc crystals were implanted both at the proximal and distal region of the flaps (Fig 7b).

Basal pO_2_ values recorded before surgery were similar in db/db (proximal site: 18.4 ± 1.3 mmHg / distal site: 18.3 ± 1.1 mmHg) and db/+ (proximal site: 20.0 ± 0.6 mmHg / distal site: 17.7 ± 0.7 mmHg) mice, and consistent with pO_2_ values measured in the unwounded subcutaneous tissue in db/db (20.7 ± 1.2 mmHg) and in non-diabetic (18.8 ± 1.2 mmHg) mice (Fig 7a). One day after surgery, a drop in pO_2_ was observed in the 2 regions of the flaps in both diabetic (proximal site: 9.4 ± 0.2 mmHg / distal site: 10.5 ± 0.3 mmHg) and non-diabetic (proximal site: 8.8 ± 0.5 mmHg / distal site: 9.2 ± 0.6 mmHg) mice. In diabetic mice, the pO_2_ remained low at proximal and distal sites until the end of the monitoring (10.0 ± 0.4 mmHg and 10.9 ± 0.3 mmHg at day 3, 14.0 ± 0.9 mmHg and 15.2 ± 1.7 mmHg at day 7, 10.5 ± 1.6 mmHg and 10.3 ± 1.9 mmHg at day 11). Macroscopically, diabetic flaps were necrotic on a large area (Fig 7c). On the contrary, in non-diabetic mice, the pO_2_ increased to reach values of 12.2 ± 1.3 mmHg and 14.9 ± 1.7 mmHg at day 3, 21.3 ± 2.0 mmHg and 17.4 ± 2.7 mmHg at day 7 for the proximal and distal regions, respectively. Macroscopically, flaps in non-diabetic mice healed with only little or no necrosis (Fig 7c).

### Intravital microscopy—microangiopathies

**Fig 8:**
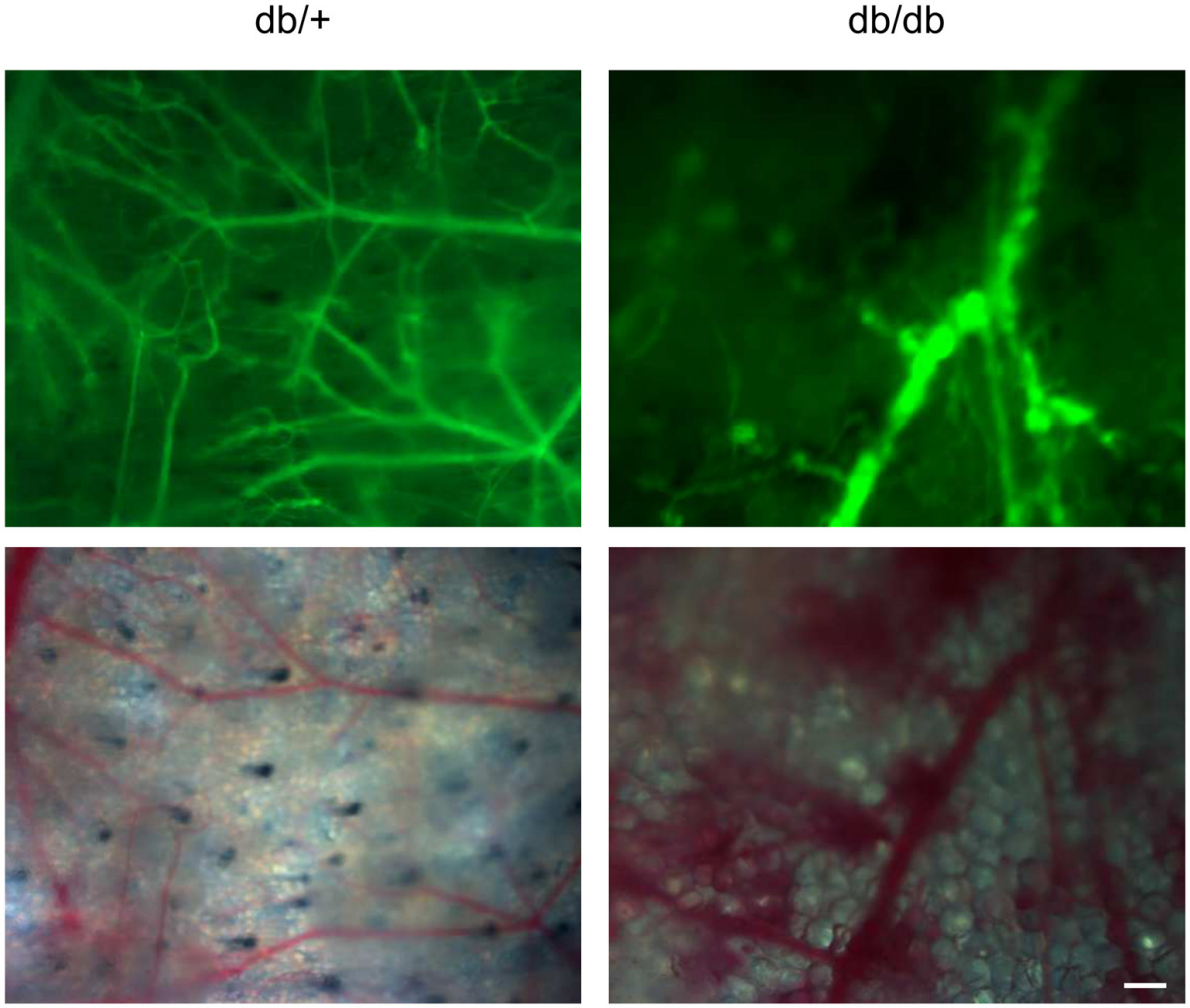
Db/db mice exhibit microvascular defects. Skin microvascular network was observed by intravital microscopy in db/+ (left) and db/db (right) mice. Images were acquired 10 minutes after a FITC-dextran injection in the tail vein. Top: fluorescence images, bottom: visible light images (scale bar = 200 μm).

Microangiopathies were evaluated by intravital microscopy. In the non-diabetic skin (Fig 8, left), a well-organized microvasculature was observed. In the diabetic skin, the microvascular network was disorganized and fewer vessels were observed compared to the non-diabetic skin (Fig 8, right). Moreover, in diabetic mice, hemorrhages were observed at the extremity of some vessels.

## Discussion

Despite the important role of oxygen in wound healing, wound oxygenation measurement is still challenging. EPR oximetry is a technique that allows absolute pO_2_ measurements that can be repeated at the same site over long periods of time, up to at least 5 years [20]. However, to date, it has never been used in the particular context of wound healing in diabetic models. This was the purpose of the present study. We showed that LiPc crystals, used as the oxygen sensor, allowed repeated and reliable pO_2_ measurements in the normal subcutaneous tissue. Indeed, the stability of the pO_2_ measurements using this oxygen sensor was verified in normal conditions, in the unwounded subcutaneous tissue. Also, a good response to hyperoxic and hypoxic challenges was observed 1 month after implantation in the subcutaneous tissue. The pertinence of wound pO_2_ measurements by EPR oximetry was determined using 2 wound models, a full-thickness excisional skin wound and a pedicled skin flap, in type 2 genetically induced diabetic db/db mice in which we observed impaired wound healing, in accordance with the literature [64–66]. In the flap model, reliable longitudinal pO_2_ measurements were obtained during the wound healing process. First, as expected, an important and sharp decrease in pO_2_ was observed one day after wounding. Indeed, in this model, the oxygen supply is dramatically decreased at the distal part of the flap as it depends only on the base of the pedicle, whereas vessels coming from the other sides are disrupted by the surgical incision and dissection [67]. Interestingly, the pO_2_ variations measured during the healing process were correlated with the macroscopic aspect of the lesion. In diabetic db/db mice, consistent with the massive necrosis observed in all animals at 7 days post wounding and microvascular defects (decrease in vessels density, unorganized vascular network, and hemorrhages observed by intravital microscopy), sustained hypoxia was evident during the entire pO_2_ monitoring period. In comparison, we observed a progressive reoxygenation of the flap linked to complete recovery in non-diabetic control db/+ mice. In the other wound model that was investigated, excisional wounds, positioning the oxygen sensor appropriately was more challenging. Contrary to the flap in which the oxygen sensor was inserted in the skin tissue undergoing flap surgery, excisional wounds are characterized by a complete removal of the wounded skin tissue. The oxygen sensor was consequently placed under the remaining thin fascia in the wound bed. When the oxygen sensor was located at the center of excisional wounds, in the wound bed, we observed significantly high pO_2_ values early after wounding that were not in agreement with the hypoxic state classically described at the early stage of wound healing [5]. Indeed, the disruption of blood vessels induced by wounding normally restricts oxygen supply to the wounded site and the increased metabolic activity of cells implicated in wound healing leads to an increase in oxygen consumption rate [5]. But it appeared that placing the oxygen sensor at this superficial position in the wound led to an over-estimation of the pO_2_ due to the influence of the surrounding atmospheric air on pO_2_ measurements, even if the sensor was laid under the fascia and the wound covered by a dressing. Placing LiPc crystals in the skin tissue at the edges of excisional wounds limited the influence of atmospheric oxygen. Indeed, in the skin tissue, we didn’t observe any difference in pO_2_ measurements when varying the oxygen concentration (0, 21 and 95%) in the surrounding atmosphere (data not shown). However, after wounding, we did not observe periods of hypoxia in this open wound model. Consequently, to reproduce hypoxia episodes, pedicled skin flap models are more appropriate.

A limitation linked to the EPR measurements is inherent to the size of LiPc crystals used in the study that was rather large compared to the mouse skin thickness. The length of the crystals was about hundreds of μm to 1 mm, limiting somewhat spatial measurement accuracy. It was in fact impossible to differentiate pO_2_ values in the different parts of the mouse skin (dermis and epidermis) and it allowed only determining a mean pO_2_ value at the implantation site in a volume of approximately 0.1 to 1 mm^3^.

Also, in order to increase the sensitivity of measurements, several crystals were implanted in the tissue. The sensor response to oxygen was not affected by the use of several crystals. Nevertheless, as measurements reflect the mean pO_2_ at the implantation site, the spatial accuracy will be lower when several crystals are used, instead of a single one, but only if a spatial dispersion of the crystals has occurred.

It must also be pointed out that the form of LiPc crystals is an important factor, as oxygen sensitivity is dependent on the structure of the crystal [68, 69]. In this study, we carefully implanted LiPc in the tissue in order not to break crystals and avoided the implantation of LiPc as a crushed powder. The aspect of LiPc crystals was verified at the end of the experiment, and the integrity of the sensors was confirmed.

Apart from the limitations attributed to the wound model and the oxygen sensor, it appeared that the choice of the animal model presenting wound healing impairment was not so straightforward. Contrary to the db/db mouse model, we did not observe any significant healing impairment in the STZ-induced diabetic mice tested. This was quite surprising as the type 1 STZ-induced diabetic model is classically used in impaired wound healing studies [70]. To allow mice to develop chronic complications of diabetes like microangiopathies, a delay of 4, 5 and 7 weeks was allowed between the occurrence of the diabetic state (confirmed by glycaemia measurements) and the wound surgery. This is the range classically mentioned in wound healing studies using this model [71, 72]. Nevertheless, even 7 weeks after the occurrence of the diabetic state, only a slight effect of diabetes was observed on wound healing kinetics. A significant effect should have been observed when the latency time between the occurrence of diabetes and wounding was longer than 7 weeks (for instance 8 to 12 weeks). An even longer delay was not tested for logistical reasons. So, the STZ-induced diabetic mice were excluded from the study and the db/db mice were selected for the wound oximetry study.

In conclusion, this study established that EPR oximetry using LiPc crystals as the oxygen sensor is applicable to monitor tissue pO_2_ during wound healing in acute and chronic wounds, in normal and diabetic animals. Nonetheless, the technique is limited for measurements in a skin flap and cannot be applied to excisional wounds in which diffusion of atmospheric oxygen significantly affects the measurements. Using the flap model, this technique could be used to evaluate the effects of pharmacological treatments on wound oxygenation and healing.

## Supporting Information

S1 Fig PO_2_ calibration curve performed with LiPc crystals. Line width (LW) in Gauss (G) of the EPR spectrum in function of the pO_2_. Results are expressed as mean ± standard deviation (n = 5).
(TIF)
