# Supplementary figures and images for: Application of Electron Paramagnetic Resonance (EPR) Oximetry to Monitor Oxygen in Wounds in Diabetic Models
Source: PLoS One. 2015 Dec 14;10(12):e0144914. doi: 10.1371/journal.pone.0144914 (PMC4679295; doi:10.1371/journal.pone.0144914)

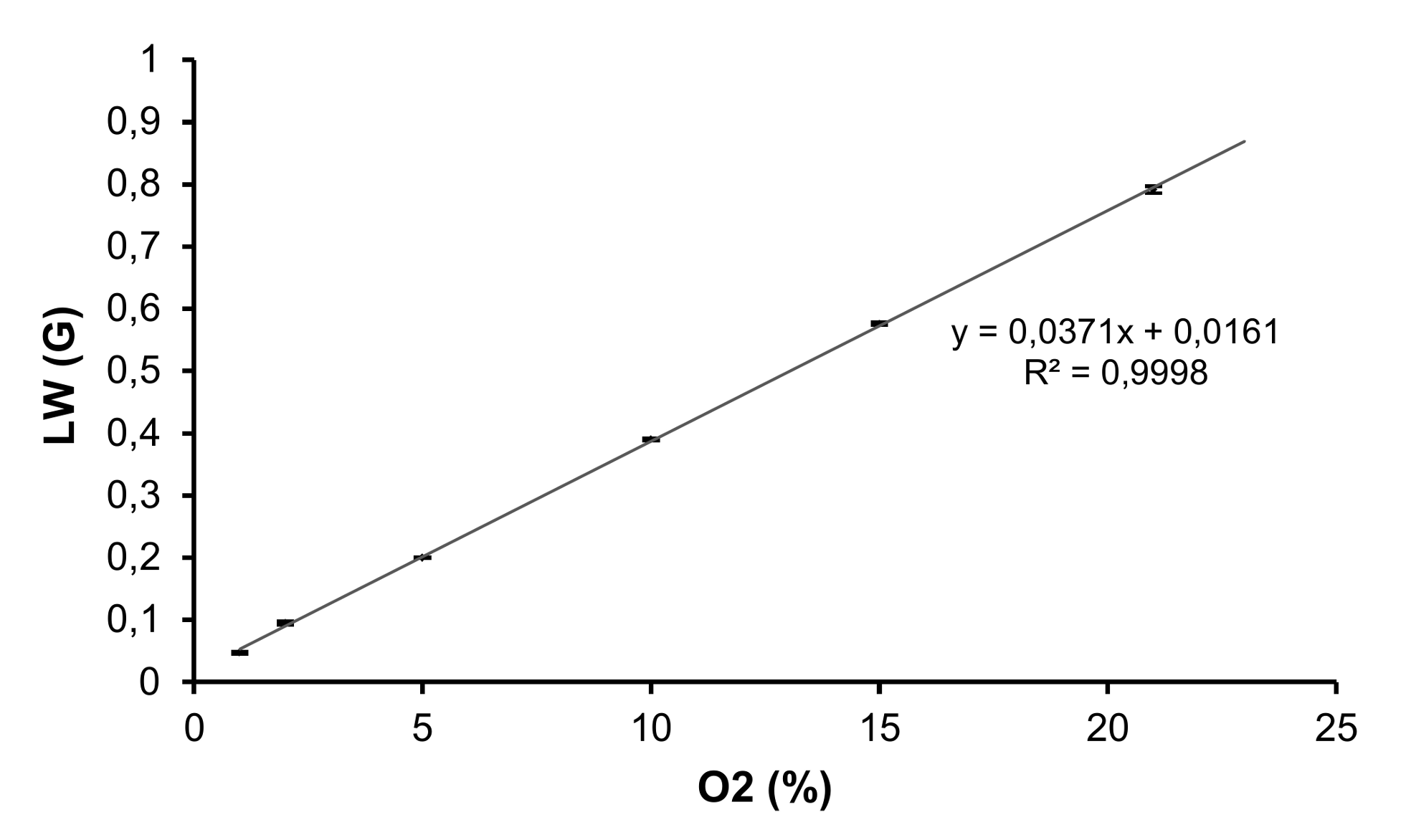

Supplement: S1 Fig — Line width (LW) in Gauss (G) of the EPR spectrum in function of the pO2. Results are expressed as mean ± standard deviation (n = 5). (TIF) [file pone.0144914.s001.tif]
